# Diacylglycerol Lipase α Knockout Mice Demonstrate Metabolic and Behavioral Phenotypes Similar to Those of Cannabinoid Receptor 1 Knockout Mice

**DOI:** 10.3389/fendo.2015.00086

**Published:** 2015-06-02

**Authors:** David R. Powell, Jason P. Gay, Nathaniel Wilganowski, Deon Doree, Katerina V. Savelieva, Thomas H. Lanthorn, Robert Read, Peter Vogel, Gwenn M. Hansen, Robert Brommage, Zhi-Ming Ding, Urvi Desai, Brian Zambrowicz

**Affiliations:** ^1^Lexicon Pharmaceuticals, Inc., The Woodlands, TX, USA

**Keywords:** mouse knockout models, diacylglycerol lipase gene, cannabinoid receptor 1 gene, obesity, endocannabinoids, 2-arachidonoylglycerol, anxiety, depression

## Abstract

After creating >4,650 knockouts (KOs) of independent mouse genes, we screened them by high-throughput phenotyping and found that cannabinoid receptor 1 (Cnr1) KO mice had the same lean phenotype published by others. We asked if our KOs of DAG lipase α or β (*Dagla* or *Daglb*), which catalyze biosynthesis of the endocannabinoid (EC) 2-arachidonoylglycerol (2-AG), or *Napepld*, which catalyzes biosynthesis of the EC anandamide, shared the lean phenotype of *Cnr1* KO mice. We found that *Dagla* KO mice, but not *Daglb* or *Napepld* KO mice, were among the leanest of 3651 chow-fed KO lines screened. In confirmatory studies, chow- or high fat diet-fed *Dagla* and *Cnr1* KO mice were leaner than wild-type (WT) littermates; when data from multiple cohorts of adult mice were combined, body fat was 47 and 45% lower in *Dagla* and *Cnr1* KO mice, respectively, relative to WT values. By contrast, neither *Daglb* nor *Napepld* KO mice were lean. Weanling *Dagla* KO mice ate less than WT mice and had body weight (BW) similar to pair-fed WT mice, and adult *Dagla* KO mice had normal activity and VO_2_ levels, similar to *Cnr1* KO mice. Our *Dagla* and *Cnr1* KO mice also had low fasting insulin, triglyceride, and total cholesterol levels, and after glucose challenge had normal glucose but very low insulin levels. *Dagla* and *Cnr1* KO mice also showed similar responses to a battery of behavioral tests. These data suggest: (1) the lean phenotype of young *Dagla* and *Cnr1* KO mice is mainly due to hypophagia; (2) in pathways where ECs signal through Cnr1 to regulate food intake and other metabolic and behavioral phenotypes observed in *Cnr1* KO mice, Dagla alone provides the 2-AG that serves as the EC signal; and (3) small molecule Dagla inhibitors with a pharmacokinetic profile similar to that of Cnr1 inverse agonists are likely to mirror the ability of these Cnr1 inverse agonists to lower BW and improve glycemic control in obese patients with type 2 diabetes, but may also induce undesirable neuropsychiatric side-effects.

## Introduction

There are an estimated 500 million obese people worldwide (1). This obesity epidemic is a major contributor to the dramatic increase in prevalence of type-2 diabetes (T2D) and the rise in diabetes complications (2). Unfortunately, efforts to induce weight loss by encouraging changes in dietary intake and physical activity alone have not reversed the obesity epidemic; this suggests that combining these behavioral modifications with a safe and effective anti-obesity drug regimen is the approach most likely to result in meaningful and sustained loss of body fat (3). Although three new anti-obesity drugs were recently approved in the United States, each has significant safety issues that are being evaluated in post-marketing studies (3). There is a need for additional anti-obesity drugs to complement and/or replace those that are currently approved.

The endocannabinoid (EC) system has long been linked to the regulation of food intake and energy balance (4). The primary psychoactive cannabinoid, Δ^9^-tetrahydrocannabinol (THC), signals through the G-protein-coupled receptors Cnr1 and Cnr2, also known as CB1 and CB2, respectively (5). The two major endogenous ligands or ECs that signal through these receptors are 2-arachidonoylglycerol (2-AG) and *N*-arachidonoylethanolamine (AEA or anandamide). The final enzymatic step in the synthesis of 2-AG is catalyzed by either diacylglycerol lipase α or β (Dagla or Daglb); by contrast, a number of enzymes, including *N*-acylphosphatidylethanolamine-hydrolyzing phospholipase D (Napepld), catalyze the final step in the synthesis of AEA (6–9). Cnr1 is highly and widely expressed in central nervous system (CNS) neurons, including neurons that regulate energy balance, and is also expressed in peripheral organs important for metabolic control; by contrast, Cnr2 is expressed primarily by cells involved in regulating immune function (10). Dagla is much more highly expressed in CNS neurons than in peripheral tissues with CNS expression mirroring that of Cnr1, Daglb is widely expressed but with much lower CNS expression than Dagla, and Napepld is widely expressed with highest expression in the CNS (6, 11). In the CNS, Dagla and Daglb demonstrate dendritic expression, consistent with the model that ECs, and particularly 2-AG, produced by post-synaptic cells inhibit presynaptic release of excitatory or inhibitory neurotransmitters by signaling through Cnr1 on presynaptic terminals (6, 12).

Food intake can be stimulated by oral delivery of THC, and by injections of either 2-AG or AEA, and this effect is blocked by the Cnr1 inverse agonist rimonabant (13–16). Further evidence that Cnr1 mediates this feeding response is provided by studies showing that hypophagia and decreased body weight (BW) are found in *Cnr1* knockout (KO) mice and in mice chronically treated with rimonabant (13, 17–21). Long-term rimonabant treatment induced weight loss not only in rodent models but also in obese humans studied in multiple clinical trials (22–24). Unfortunately, neuropsychiatric side-effects, which were likely on-target considering the role of ECs in neural pathways regulating a wide range of emotional behaviors, ultimately led to withdrawal of rimonabant from the market (24–26).

In an effort to identify potential drug targets, Lexicon Pharmaceuticals performed a high-throughput phenotypic screen on >4,650 KOs of independent genes encoding druggable proteins (27–31). As part of this phenotypic screen, body fat was measured in cohorts of chow-fed mice from 3,651 KO lines. Among KO lines identified with potential body fat phenotypes, *Cnr1* KO mice had a lean phenotype, confirmed using additional cohorts of mice (30), that was consistent with the lean phenotype of *Cnr1* KO mice reported by others (17, 20). Although *Dagla*, *Daglb*, and *Napepld* KO lines have been generated (9, 32, 33), their body fat levels have not been reported. Because these three proteins are potentially druggable enzymes, *Dagla*, *Daglb*, and *Napepld* KO lines were phenotyped in our screen. We chose to review the screen results for body fat from these KO lines to determine if any or all shared the lean phenotype of *Cnr1* KO mice, and if so to examine them in greater detail to determine how closely other phenotypic characteristics mirrored those of *Cnr1* KO mice, including behavioral phenotypes that suggest the possibility of on-target neuropsychiatric side-effects which would likely preclude developing inhibitors of these enzymes as anti-obesity drugs for humans.

## Materials and Methods

### Generation of KO mice

*ApoE* KO mice were obtained from Taconic (catalog no. APOE-M, Taconic Biosciences, Hudson, NY, USA). All other KO mice were generated at Lexicon Pharmaceuticals on a 129S5/SvEvBrd x C57BL/6-Tyr*^c-Brd^* hybrid background. Our approach to knocking out and phenotyping mouse orthologs of the potentially druggable genes in the human genome is published (27–31). *Cnr1* KO, *Dagla* KO, *Daglb* KO, and *Dagla*/*Daglb* double KO (*dagla/b* DKO) mice have been described (30, 33). Mice heterozygous for both *Dagla* and *ApoE* were bred to generate *Dagla*/*ApoE* DKO mice. *Napepld* KO mice were generated by gene trapping; methods for gene trapping in embryonic stem cells, identifying trapped genes using OmniBank Sequence Tags, characterizing retroviral gene-trap vector insertion sites, and RT-PCR analysis of KO and wild-type (WT) transcripts are published (34). Briefly, a retroviral gene-trap vector was used to produce OmniBank clone OST429065, which contains an intron 3 insertion that truncates the *Napepld* gene product immediately after the second coding exon; this clone was used to generate *Napepld* KO mice (Figure S1 in Supplementary Material). Genotyping was performed on tail DNA as described previously (34).

### Mouse care and study

All studies were performed in strict accordance with the recommendations in the Guide for the Care and Use of Laboratory Animals of the National Institutes of Health. The protocols for all studies were approved by the Lexicon Institutional Animal Care and Use Committee (OLAW Assurance Number, A4152-01; AAALAC International Accreditation Number, 001025). General methods for mouse care have been described (34). Mice were fed either standard rodent chow (3.56 kcal/g; 9F 5020, Purina, St Louis, MO, USA), low-fat diet (LFD) containing 10% kcal from fat (3.85 kcal/g; D12450B, Research Diets, New Brunswick, NJ, USA), high-fat diet (HFD) containing 45% kcal from fat (4.73 kcal/g; D12451, Research Diets), or western diet (4.7 kcal/g; D12079B; Research Diets). Pair-feeding and food consumption studies were performed as described previously (35).

### Body composition determinations

Body composition was measured using either dual energy x-ray absorptiometry (DEXA; PIXImus, InsideOutside Sales, Fitchburg, WI, USA) or quantitative magnetic resonance (QMR, ECHO Medical Systems, Houston, TX, USA) technologies as described previously (30). For our phenotypic screen, body fat was analyzed on 14-week-old mice by DEXA and data presented as described previously (30). Briefly, for each KO line, mean KO % body fat/mean WT littermate % body fat was calculated for both male and female mice, and then these male and female values were averaged, yielding a normalized % body fat value. For most KO lines, four male KO, two male WT, four female KO, and two female WT mice were analyzed. Lines with fewer than four KO mice or fewer than three WT mice were excluded. For lines with uneven distributions of male and female mice, values for the normalized % body fat calculation were weighted to take into account the actual number of mice analyzed. For X-linked lines, only male data were used.

### Stool analysis

Group-housed KO and WT mice were fed HFD containing green dye to aid in stool identification. Each cage of mice underwent 24-h stool collections for three consecutive days. After each 24-h stool collection was weighed, lipids were extracted over 24 h using distilled acetone in a Soxhlet apparatus. After removal of residual acetone by evaporation, each stool collection was reweighed, with the difference in weights representing the amount of acetone-extractable fat.

### Measurements of physical activity and VO_2_

Mice were individually placed in Oxymax chambers (Oxymax, Columbus Instruments, Columbus, OH, USA) and allowed to acclimate overnight. Starting at 10:00 a.m. the following day, each mouse had VO_2_ and physical activity measured over the next 24 h as described previously (35). Mice were also studied in a second system that measures physical activity, the ER-4000 physiological measurement system (Mini Mitter, Bend, OR, USA). In these studies, mice first had E-mitter transponders surgically implanted into their peritoneal cavity; after a 3-day recovery period, mice were placed in individual cages within range of an ER-4000 receiver, which measures activity by sensing the strength of the signal received from the E-mitter. Activity data for each mouse were collected in 10 min intervals, and averaged per hour, for a period of 3 weeks using the VitalView Data Acquisition System (Mini Mitter).

### Blood sample analysis

Unless stated otherwise, blood was obtained from fed mice by retro-orbital bleed and serum was assayed for glucose, total cholesterol, total triglycerides (TGs), alanine aminotransferase (ALT), and aspartate aminotransferase (AST) by Cobas Integra 400 analyzer (Roche Diagnostics, Indianapolis, IN, USA) as described previously (34).

### Oral glucose tolerance tests

Oral glucose tolerance tests (OGTTs) were performed on conscious, unanesthetized mice. After an overnight fast, mice were bled from the retro-orbital plexus at baseline and then received 2 g/kg glucose by oral gavage. Whole-blood samples obtained from the retro-orbital plexus at 0, 30, and 60 min were directly assayed for glucose levels by ACCU-CHEK Aviva glucometer (Roche, Indianapolis, IN, USA); serum obtained at 0 and 30 min was used to measure insulin levels (Ultra Sensitive Rat Insulin ELISA Kit, Cat. 90060; Crystal Chem, Downers Grove, IL, USA).

### Pathology

Livers obtained from 20- to 24-week-old WT, *Dagla* KO, *ApoE* KO, and *Dagla/ApoE* DKO littermate mice fed western diet since weaning were prepared for histopathologic examination as described previously (35). Liver sections were interspersed during reading by a pathologist to reduce bias, and were given semi quantitative scores of 0–4, without knowledge of genotype, for degree and extent of fatty cytoplasmic vacuolation (steatosis) and for number and extent of small histiocytic and mixed inflammatory foci replacing hepatic parenchyma (inflammation) (36).

### Behavioral testing

*Dagla* and *Cnr1* KO mice were studied in tail suspension and forced swim tests developed to screen antidepressant compounds (37), open field and platform tests developed to screen anxiolytic compounds (38), the hot plate test used to assess nociceptive pain (39), and the marble burying test used to screen compounds for possible effects on anxiety, depression, and the repetitive behavior of obsessive compulsive disorder (40). Our protocols for tail suspension, forced swim, open field, marble burying, and hot plate tests are published (41), as is our protocol for the platform test (38).

### Statistics

Data are presented as mean ± SD. Unless stated otherwise, comparisons between two groups were analyzed by unpaired Student’s *t*-test, and comparisons among three or more groups were analyzed by one-way ANOVA, with *post hoc* analysis performed using the Bonferroni correction. For all behavioral tests, for body fat analysis from the *Dagla/b* DKO study, and for liver histopathology and body composition analyses from the *Dagla/ApoE* DKO study, two-way ANOVA was employed. All statistical tests were performed using PRISM 4.03 (GraphPad). Differences were considered statistically significant when *P* < 0.05.

## Results

**Figure 1:**
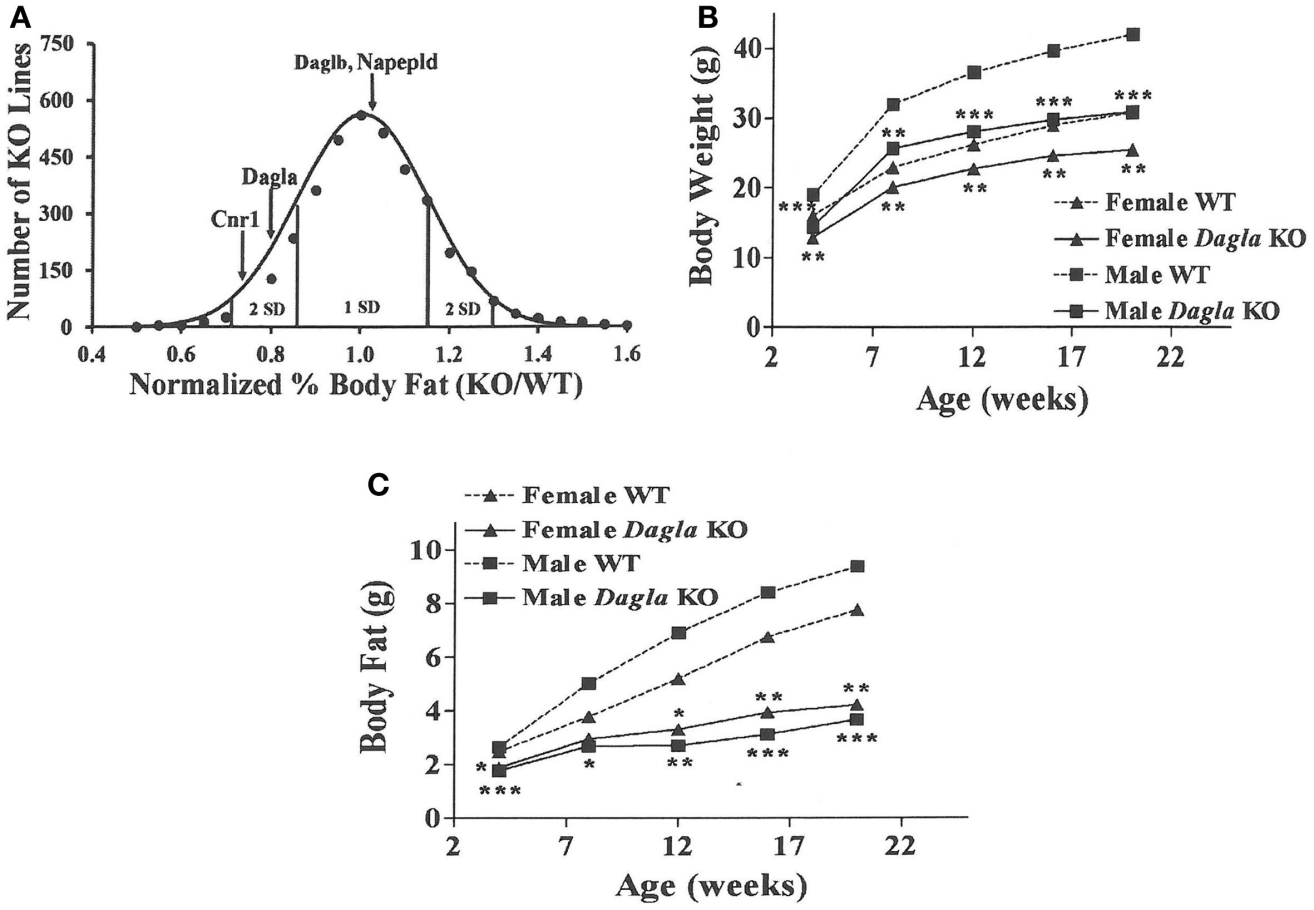
**Chow-fed *Dagla* KO mice are lean**. **(A)** Histogram of normalized% body fat for 3,651 knockout lines maintained on chow diet. Body composition analyses performed by DEXA on 14-week-old male mice fed chow diet from weaning were used to calculate normalized % body fat for each knockout line as described in Materials and Methods. The mean normalized % body fat value was 1.08 ± 0.15; the distribution was normal based on Kolmogorov–Smirnov testing (*P* < 0.01). Solid points indicate actual numbers of knockout lines. Curved line shows the calculated curve; the range for 1 and 2 SD from the mean are indicated by lines located below the curve, and values for *Cnr1*, *Dagla*, *Daglb*, and *Napepld* KO mice are indicated by lines shown above the curve. **(B)** Time course for body weight of chow-fed *Dagla* KO mice and their WT littermates. **(C)** Time course for body fat measured by QMR of chow-fed *Dagla* KO mice and their WT littermates. For each data point in **(B,C)**, *N* = 7–15 mice. KO mice different from WT mice of the same age and gender, *P < 0.05, **P < 0.01, ***P < 0.001.

Similar to *Cnr1* KO mice, *Dagla* KO mice were lean relative to WT littermates in our high-throughput phenotypic screen of 3,651 KO lines maintained on chow diet; by contrast, neither *Daglb* nor *Napepld* KO mice were lean in this screen (Figure 1A). *Dagla* mice had normal Mendelian distribution at weaning (923 WT, 1882 Het, 971 KO) and appeared healthy, similar to *Cnr1* (378 WT, 717 Het, 318 KO), *Daglb* (173 WT, 332 Het, 169 KO), and *Napepld* (77 WT, 150 Het, 82 KO) mice. To confirm the lean phenotype observed during screening, additional cohorts of chow-fed male and female *Dagla* KO mice were studied and were found to have significantly decreased BW, body fat, and lean body mass (LBM) at weaning and through 20 weeks of age (Figures 1B,C).

**Figure 2:**
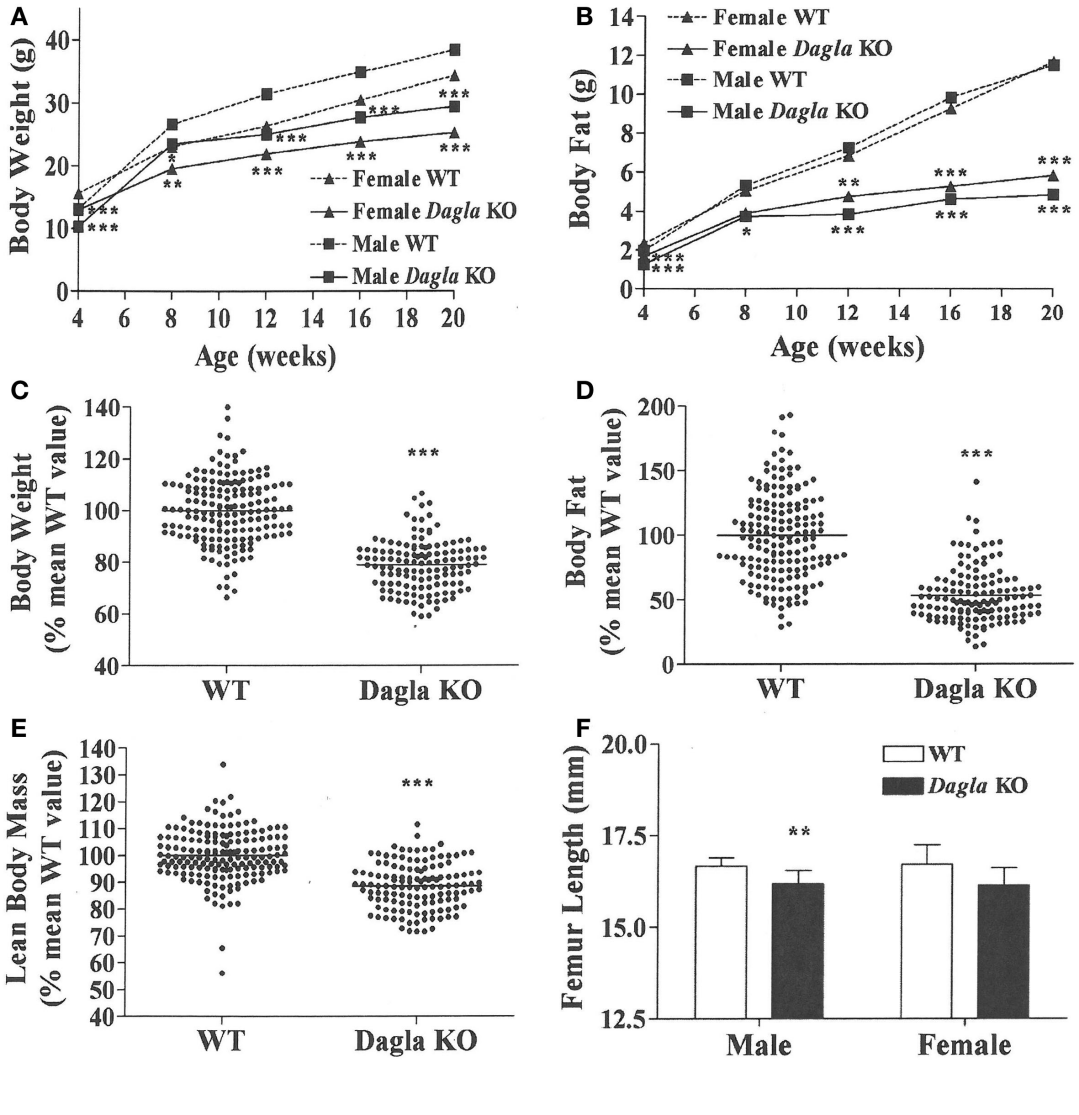
***Dagla* KO mice are lean on various diets**. **(A)** Time course for body weight of *Dagla* KO mice and their WT littermates fed HFD. **(B)** Time course for body fat measured by QMR of *Dagla* KO mice and their WT littermates fed HFD. For **(A,B)**, *N* = 12–15 mice for each data point. Normalized data for **(C)** body weight, **(D)** body fat measured by QMR, and **(E)** LBM measured by QMR on 16-week-old *Dagla* KO mice (*N* = 131) and their WT littermates (*N* = 169) fed various diets. **(F)**. Length of femurs from *Dagla* KO mice (*N* = 8 males and 6 females) and their WT littermates (*N* = 9 males and 8 females). KO mice different from WT mice of the same age and gender, *P < 0.05, **P < 0.01, ***P < 0.001.

**Figure 3:**
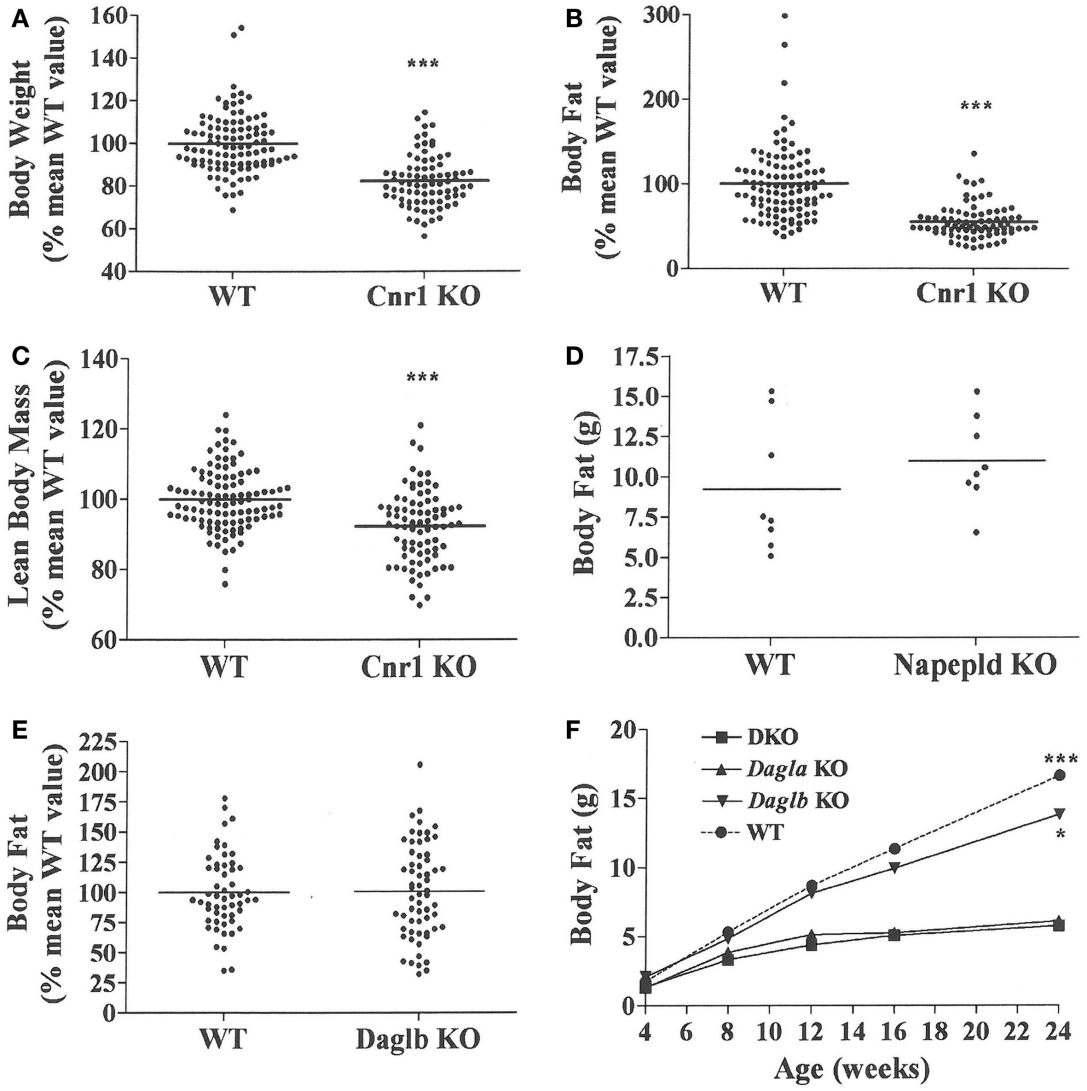
**Presence of a lean phenotype in *Cnr1* KO mice but not *Napepld* or *Daglb* KO mice**. Normalized data for **(A)** body weight, **(B)** body fat measured by QMR and **(C)** LBM measured by QMR on 16- to 32-week-old *Cnr1* KO mice (*N* = 80) and their WT littermates (*N* = 102). **(D)** Body fat measured by QMR on 8 male *Napepld* KO mice and 8 male WT littermates at 12 weeks of age. **(E)** Normalized body fat measured by QMR on 61 *Daglb* KO mice and 54 WT littermates at 15–16 weeks of age. For **(A–E)**, KO mice different from WT mice, ***P < 0.001. **(F)** Time course for body fat measured by QMR on female *Dagla* KO, *Daglb* KO, *Dagla/b* DKO, and WT littermates (*N* = 6–8 mice/group). When analyzed by one-way ANOVA, different from *Dagla* KO and DKO mice, *P < 0.05, ***P < 0.001. When analyzed by two-way ANOVA, *Dagla* KO mice different from WT mice, *P* < 0.001; there was no interaction between *Dagla* KO and *Daglb* KO on body fat.

*Dagla* KO mice fed HFD from weaning developed significantly decreased BW, body fat, and LBM relative to WT littermates (Figures 2A,B). When we combined QMR data obtained at 15–17 weeks of age from multiple chow- and HFD-fed cohorts, we found that BW, body fat, and LBM were significantly decreased by 21, 47, and 11%, respectively, in 131 *Dagla* KO mice compared to 169 WT littermates (Figures 2C–E; Table S1 in Supplementary Material); because normalized data from male and female KO mice were comparable, they were combined for this analysis. The lean phenotype persisted with age, and was still present in *Dagla* KO mice aged more than 1 year (Table 1). The body composition phenotype of *Dagla* KO mice was confirmed by DEXA analysis (Table S2 in Supplementary Material), which also revealed a slightly decreased bone mass for 29-week-old *Dagla* KO mice that was probably appropriate for their slightly smaller skeletal frame as demonstrated by femur length measurements (Figure 2F). The lean phenotype of *Dagla* KO mice is quite similar to that observed in our *Cnr1* KO mice (30). In an update of our *Cnr1* KO data, we combined QMR measurements obtained at 16–32 weeks of age from multiple chow- and HFD-fed cohorts and found that BW, body fat, and LBM were significantly decreased by 18, 45, and 8%, respectively, in 80 *Cnr1* KO mice compared to 102 WT littermates (Figures 3A–C; Table S3 in Supplementary Material); because normalized data from these male and female KO mice were comparable, they were combined for this analysis. By contrast, neither *Napepld* nor *Daglb* KO mice raised on HFD showed decreased body fat at 15 weeks of age (Figures 3D,E). In addition, *DAGLa/b* DKO mice showed no enhancement of the low body fat phenotype over that observed in *DAGLa* KO mice (Figure 3F); these mice appeared healthy at weaning, and their Mendelian distribution was normal.

**Table 1:** **Body composition by QMR of *Dagla* KO and WT mice at >1 year of age**.

Cohort	Genotype	*N*	Age (months)	Body weight (g)	Body fat (g)	% Body fat	LBM (g)
2-M	WT	9	14	55.8 ± 8.4	19.8 ± 4.8	35.0 ± 5.5	36.0 ± 4.5
2-M	KO	6	14	41.9 ± 5.8*	13.1 ± 6.1*	35.0 ± 7.3	28.8 ± 4.2**
2-F	WT	6	14	35.8 ± 7.7	11.1 ± 7.4	29.0 ± 12.9	24.7 ± 2.6
2-F	KO	7	14	24.5 ± 2.1**	3.9 ± 0.7*	16.0 ± 1.9*	20.6 ± 1.7**
3-M	WF	10	14	55.1 ± *6.5*	21.1 ± 4.5	37.9 ± 4.1	34.0 ± 2.5
3-M	KO	8	14	34.3 ± 6.9***	8.6 ± 4.6***	23.7 ± 8.0***	25.7 ± 2.5***
3-F	WF	10	15	40.5 ± 9.4	15.4 ± 7.7	36.3 ± 9.5	25.1 ± 2.4
3-F	KO	6	15	26.3 ± 4.2**	6.0 ± 1.3*	22.9 ± 2.7**	20.3 ± 3.2**
8-F	WT	7	15	42.4 ± 9.3	16.8 ± 7.4	38.0 ± 9.7	25.6 ± 2.3
8-F	KO	7	15	26.8 ± 2.9**	5.1 ± 1.5**	18.7 ± 3.8***	21.7 ± 1.7**

Data presented as mean ± SD; *N* = number of mice; M = male; F = female; LBM, lean body mass. KO different from WF, *P < 0.05; **P < 0.01; ***P < 0.001.

**Figure 4:**
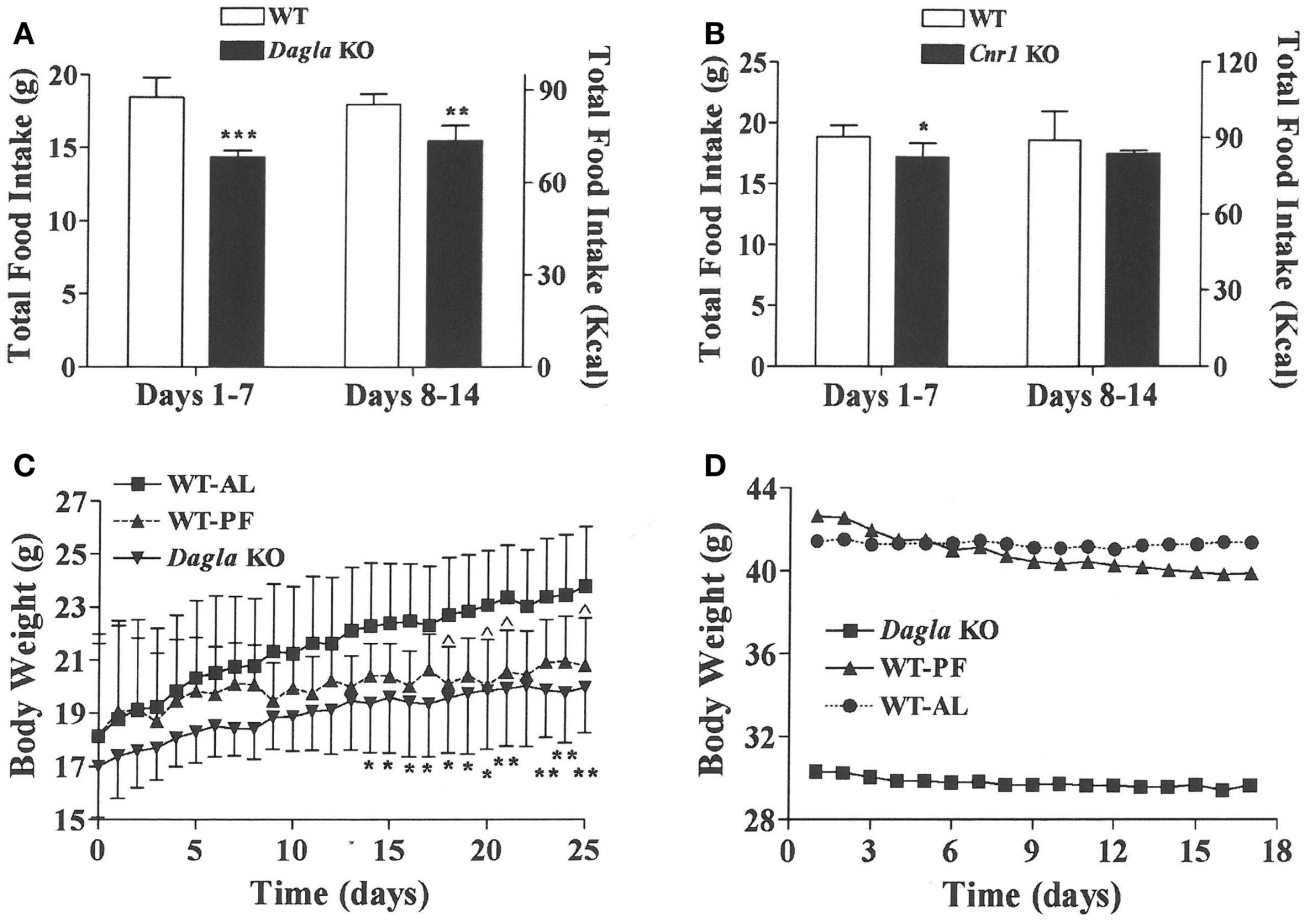
**Decreased food intake by *Dagla* and *Cnr1* KO mice at weaning**. **(A)** Intake of HFD by *Dagla* male mice (three mice/cage, five cages/genotype). **(B)** Intake of HFD by *Cnr1* male mice (two to three mice/cage, five cages/genotype). For **(A,B)**, KO mice different from WT mice, *P < 0.05, **P < 0.01, ***P < 0.001. **(C)** Body weights of ad lib-fed *Dagla* KO mice, ad lib-fed WT littermate mice (WT-AL), and WT littermate mice that were pair-fed to the *Dagla* KO mice (WT-PF). Mice were individually housed weanling female littermates fed LFD; seven mice/group. KO mice different from WT-AL mice, *P < 0.05, **P < 0.01; WT-PF mice different from WT-AL mice, ^*P* < 0.05. **(D)** Body weights of ad lib-fed *Dagla* KO mice, ad lib-fed WT littermate mice (WT-AL), and WT littermate mice that were pair-fed to the *Dagla* KO mice (WT-PF). All mice were individually housed 27-week-old male littermates fed HFD; seven mice/group.

**Figure 5:**
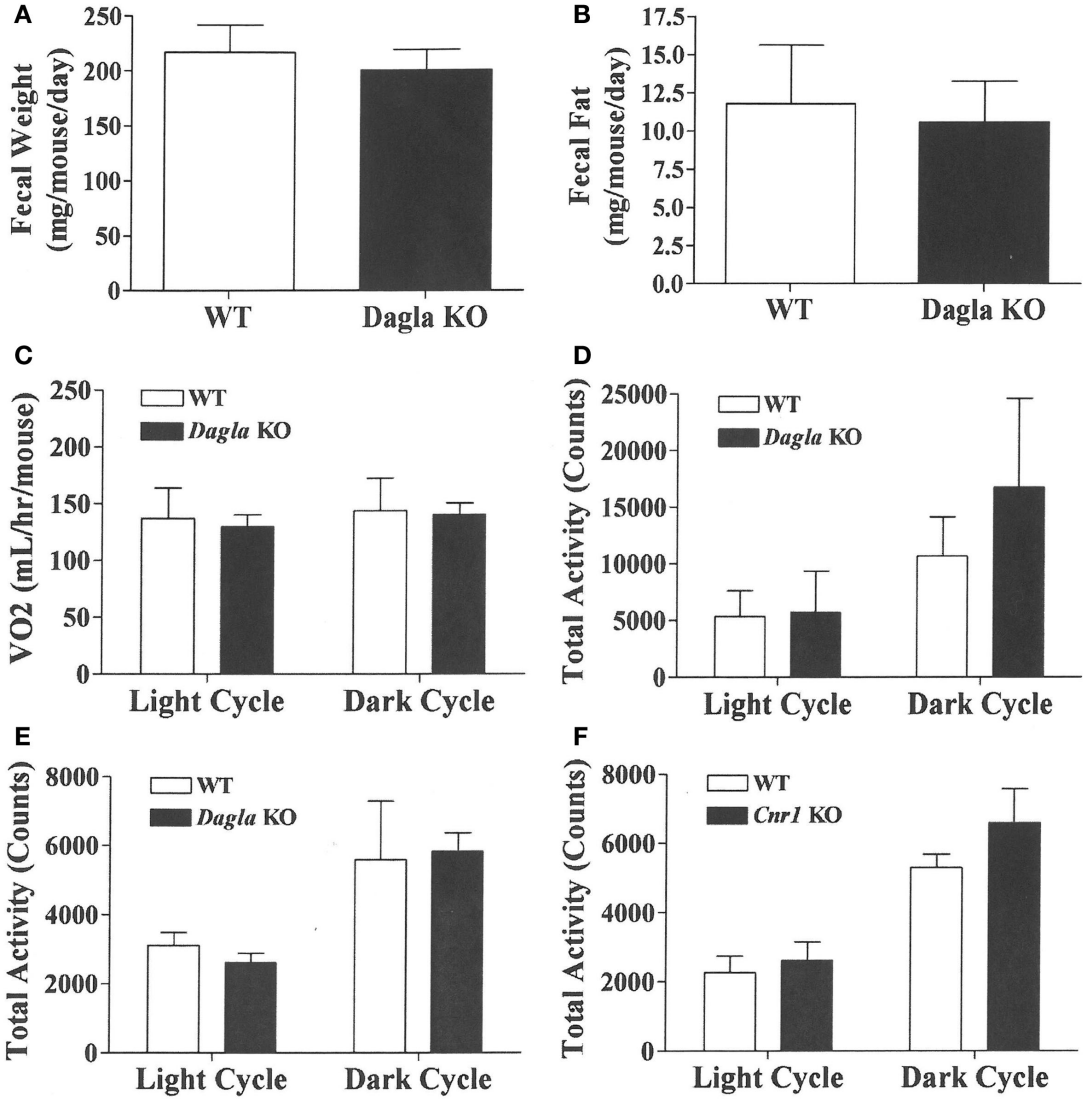
**Fecal weight, fecal fat and VO_2_ of *Dagla* KO mice, and activity levels of *Dagla* and *Cnr1* KO mice**. Fecal weight **(A)** and fecal fat **(B)** from HFD-fed, 8-week-old group-housed male *Dagla* KO (four cages) and WT littermate (five cages) mice; mice were housed three per cage. VO_2_**(C)** and total activity **(D)** levels of HFD-fed, 45-week-old female *Dagla* KO mice (*N* = 6) and their WT littermates (*N* = 8) measured in Oxymax chambers over 24 h as described in the Section ‘Materials and Methods.’ **(E)** Total activity levels of HFD-fed, 30-week-old male *Dagla* KO mice (*N* = 9) and their WT littermates (*N* = 7), and **(F)** activity levels of HFD-fed, 30-week-old male *Cnr1* KO mice (*N* = 5) and their WT littermates (*N* = 6), measured over 3 weeks in the ER-4000 physiological measurement system as described in the Section ‘Materials and Methods.’

We sought to identify the mechanism behind the low-body fat phenotype of *Dagla* KO mice. We found that *Dagla* KO mice ate significantly less than WT littermates at weaning (Figure 4A), similar to *Cnr1* KO mice (Figure 4B). A pair-feeding study, performed on female *Dagla* mice beginning at weaning, showed that pair-fed WT mice had BWs similar to *Dagla* KO mice and significantly less than WT mice fed ad lib (Figure 4C), suggesting that the lean phenotype of *Dagla* KO mice at weaning was primarily due to decreased food intake. By contrast, when we studied 27-week-old *Dagla* KO mice with an established lean phenotype, we found that WT littermates pair-fed to these *Dagla* KO mice showed an insignificant loss of BW relative to WT littermates that were fed ad lib (Figure 4D). Adult *Dagla* KO mice did not show evidence of malabsorption (Figures 5A,B) and, in addition, had VO_2_ (Figure 5C) and total activity (Figure 5D) levels that were not different from those of WT littermate controls. The total activity data were confirmed using a different technology in a separate cohort (Figure 5E). Similar to *Dagla* KO mice, *Cnr1* KO mice had total activity levels that were comparable to levels of WT littermates (Figure 5F).

**Figure 6:**
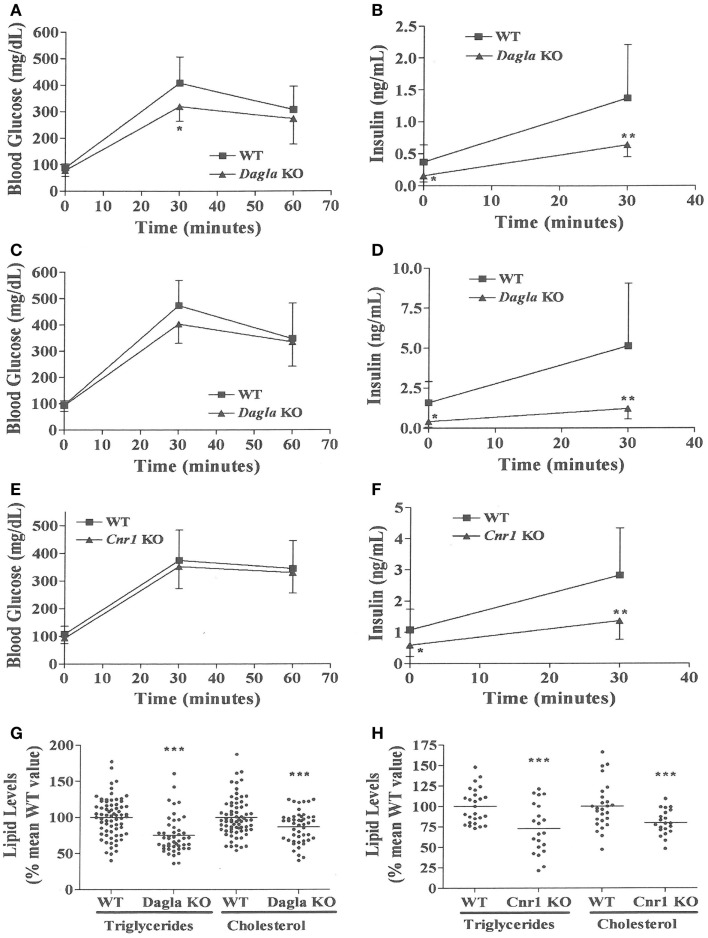
**Improved OGTT and fasting serum lipid measurements in *Dagla* and *Cnr1* KO mice**. **(A)** Glucose excursions, and **(B)** 0 and 30 min insulin levels, from an OGTT performed on HFD-fed male *Dagla* KO mice (*N* = 12) and their WT littermates (*N* = 14) at 16 weeks of age. **(C)** Glucose excursions, and **(D)** 0 and 30 min insulin levels, from an OGTT performed on and independent cohort of HFD-fed male *Dagla* KO mice (*N* = 10) and their WT littermates (*N* = 6) at 18 weeks of age. **(E)** Glucose excursions, and **(F)** 0 and 30 min insulin levels, from an OGTT performed on HFD-fed male *Cnr1* KO mice (*N* = 14) and their WT littermates (*N* = 15) at 16 weeks of age. For **(A–F)** above, KO glucose AUC and insulin levels different from WT, *P < 0.05, **P < 0.01. Normalized fasting serum triglyceride and total cholesterol levels from **(G)** adult *Dagla* KO mice (*N* = 47) and their WT littermates (*N* = 70), and **(H)** adult *Cnr1* KO mice (*N* = 21) and their WT littermates (*N* = 26); KO different from WT, ***P < 0.001.

We evaluated the effect of *Dagla* deficiency on metabolic parameters. Although *Dagla* KO mice maintained on HFD had fasting blood glucose levels that were not different from WT, and responded to an OGTT with only modest lowering of glucose excursions relative to WT values, they did exhibit significantly lower 0 and 30 min insulin levels (Figures 6A–D); thus, less insulin was required to maintain post-prandial glucose excursions in these non-diabetic mice. *Cnr1* KO mice maintained on HFD also showed no difference from WT mice in terms of fasting blood glucose and, during OGTTs, exhibited an insignificant improvement in glucose excursions associated with significantly lower 0 and 30 min insulin levels (Figures 6E,F). Fasting TG and total cholesterol levels tended to be lower in multiple cohorts of *Dagla* KO mice-fed HFD (Table S4 in Supplementary Material); when data were normalized such that mean WT values for each male and female cohort were assigned a value of 100%, and all data were then combined, TG and total cholesterol levels were significantly lower in *Dagla* KO mice, measuring 75 and 87% of WT littermate values, respectively (Figure 6G). *Cnr1* KO mice weaned onto HFD showed a similar pattern (Table S4 in Supplementary Material); when data were again normalized and combined, TG and total cholesterol levels were significantly lower in *Cnr1* KO mice, measuring 83 and 80% of WT littermate values, respectively (Figure 6H). We also studied whether *Dagla* deficiency would protect against hepatic steatosis and inflammation by placing *Dagla* KO mice, *ApoE* KO mice, *Dagla/ApoE* DKO mice, and WT littermates on Western diet from weaning (Table 2). When these mice were studied at 20–24 weeks of age, *Dagla* but not *ApoE* KO mice were lean relative to WT littermates, and histologic examination of hepatic tissue demonstrated that *Dagla* deficiency significantly protected WT mice from steatosis but not inflammation, whereas *ApoE* deficiency had no effect on either parameter in this analysis. Serum analysis showed that *ApoE* KO mice had increased TG, total cholesterol, and AST relative to WT littermates, and that the addition of *Dagla* deficiency to *ApoE* deficiency significantly lowered each of these parameters. Comparable studies were not performed using *Cnr1* KO mice.

**Table 2:** **Serum lipids, liver function/steatosis, and body fat in Dagla KO, ApoE KO, and Dagla/ApoE DKO mice-fed Western diet**.

	WT	*Dagla* KO	*ApoE* KO	*D/A* DKO
ALT, U/L	136 ± 107 (19)	137 ± 98 (13)	124 ± 94 (17)	43 ± 35 (10)
AST, U/L	87 ± 72 (19)***	83 ± 23 (13)**	176 ± 85 (17)	90 ± 49 (10)*
TG, mg/dL	82 ± 17 (19)***	84 ± 15 (13)***	308 ± 153 (17)	171 ± 71 (10)**
Chol, mg/dL	220 ± 76 (19)***	173 ± 59 (13)***	1713 ± 621 (17)	823 ± 759 (10)***
Body fat^a^, g	14.7 ± 4.2 (19)	8.3 ± 3.7 (13)^^^^^	13.4 ± *5.5* (17)	6.7 ± 4.1 (10)
Liver steatosis^b^	*2.9* ± 1.5 (5)	1.3 ± 1.0 (11)^^^^^	2.8 ± 0.5 (7)	1.4 ± 1.0 (7)
Liver inflammation^c^	1.2 ± 1.2 (5)	0.8 ± 0.8 (7)	2.0 ± 0.5 (11)	1.6 ± 0.8 (7)

*N, number of mice; TG, triglyceride; Chol, total cholesterol; D/A DKO, Dagla/ApoE double KO mice. All serum samples obtained from fed female mice at 20–24 weeks of age*.

*^a^Body fat measured by QMR at 20–24 weeks of age*.

*^b^Liver steatosis score in semi quantitative units (see Materials and Methods); higher value suggests more steatosis*.

*^c^Liver inflammation score in semi quantitative units (see Materials and Methods): higher value suggests more inflammation*.

Different from WT by two-way ANOVA, ^^^^^P < 0.001. Different from ApoE KO by one-way ANOVA, *P < 0.05, **P < 0.01, ***P < 0.001.

*Dagla* and *Cnr1* KO mice were also evaluated for their performance during a number of behavioral tests (Table 3). During the tail suspension test, neither *Dagla* nor *Cnr1* KO mice showed a significant difference in immobility time relative to WT littermates. The forced swim test showed significantly decreased immobility time in *Dagla* KO mice, while a much smaller cohort of *Cnr1* KO mice showed a strong trend (*P* = 0.07) toward a similar decrease. In the open-field test, neither *Dagla* nor *Cnr1* KO mice differed from WT littermates in total distance traveled, and both showed significantly less rearing activity than did their WT controls; by contrast, the *Cnr1* KO mice spent significantly less time than WT littermates in the center of the open field, whereas *Dagla* KO and WT littermates were not different for this parameter. This last finding was revisited in the platform test, which provides a more specific measure of anxiety-related behavior (38); *Dagla* KO mice spent significantly more time than WT mice in the lighted area, and a much smaller cohort of *Cnr1* KO and WT mice did not differ significantly for this parameter. Finally, both *Dagla* and *Cnr1* KO mice buried significantly fewer marbles during the marble burying test, and both took significantly longer than their WT littermate controls to respond to a thermal stimulus during the hot plate test. For all tests except the marble burying in *Dagla* KO mice, and the forced swim and platform tests in *Cnr1* KO mice, the findings in Table 3 were confirmed with at least one additional independent cohort of mice (data not shown).

**Table 3:** **Behavioral studies in *Dagla* and *Cnr1* KO mice**.

	*Dagla*		*Cnr1*	

Test	WT	KO	WT	KO
**Tail suspension**				
Immobility time, s	104 ± 40 (21)	129 ± 64 (16)	76 ± 42 (24)	94 ± 56 (20)
**Forced swim**				
Immobility time, s	237 ± 66 (25)	123 ± 86 (17)***	168 ± 77 (8)	84 ± 78 (6)
**Open field**				
Total distance, cm	2244 ± 783 (26)	2138 ± 766 (I8)	1724 ± 917 (25)	1265 ± 855 (24)
Time in center, s	313 ± 117 (26)	267 ± 193 (18)	346 ± 198 (25)	147 ± 108 (24)***
Rearing, *N*	51 ± 25 (26)	29 ± 26 (18)**	50 ± 43 (25)	13 ± 18 (24)***
**Platform**				
Time in light, s	75 ± 68 (26)	187 ± 77 (17)***	108 ± 97 (8)	70 ± 100 (7)
**Hot plate**				
Latency to respond, s	10.6 ± 3.6 (26)	15.3 ± 5.3 (18)**	8.6 ± 3.5 (25)	10.9 ± 3.9 (24)*
**Marble burying**				
Marbles buried, *N*	13 ± 7 (26)	6 ± 7 (18)**	10 ± 7 (25)	4 ± 5 (24)***

N, number of mice; s, seconds. Different from WT, *P < 0.05, **P < 0.01, ***P < 0.001.

*Note: Cnr1 forced swim and platform tests were analyzed by Student’s t-test because only female mice were studied. In all other groups, two-way ANOVA showed no gender × genotype interaction, so male and female data were combined*.

**Figure 7:**
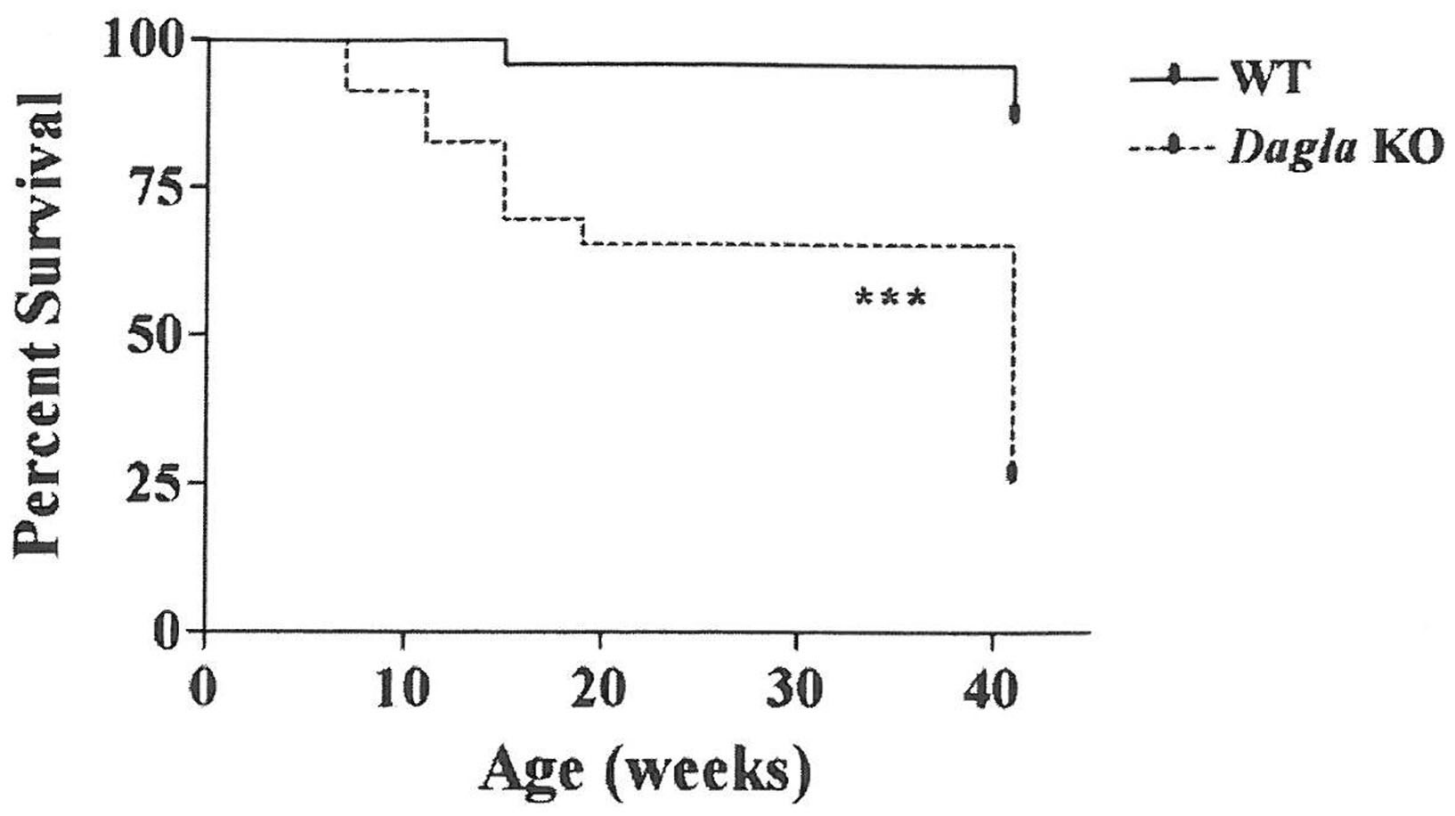
**Decreased survival of *Dagla* KO mice**. Survival curves of combined male and female *Dagla* KO mice and their WT littermates from weaning through 41 weeks of age. KO different from WT, ***P < 0.001.

Long-term survival was significantly decreased in *Dagla* KO mice (Figure 7). Despite this impaired survival, aging *Dagla* KO mice appeared healthy. In a cohort of 12 *Dagla* KO mice that were at least 1 year of age at necropsy, no pathological changes were noted in brain, heart, or other tissues examined that could explain the decreased survival of these mice (data not shown). On three separate occasions, *Dagla* KO mice undergoing QMR were observed to have spontaneous seizure activity and none of these mice survived the seizure episode, suggesting that the decreased survival was related to an underlying neurological abnormality which was not evident during histopathologic analysis of brain tissue.

## Discussion

Our *Dagla* and *Cnr1* KO mice share the same lean phenotype when raised on either chow or HFD. In fact, the similarity of their lean phenotypes was remarkable, with body fat 47 and 45% lower in *Dagla* and *Cnr1* KO mice, respectively, relative to WT littermates. By contrast, *Daglb* and *Napepld* KO mice had normal body fat stores. The lean phenotype of our *Cnr1* KO mice confirms published data (17, 20, 30). By contrast, body fat data have not been reported for *Dagla* or *Daglb* KO mice, although one study did report that BW was significantly decreased in *Dagla* KO mice and slightly decreased in *Daglb* KO mice (32). The absence of a body composition phenotype in *Napepld* KO mice is consistent with recent data indicating that this enzyme is not required for maintenance of normal CNS AEA levels (9). We also found that *Dagla* and *Cnr1* KO mice are hypophagic, consistent with past studies showing decreased feeding behavior in *Cnr1* KO mice (17, 18, 20). In our studies, decreased food intake was demonstrated in weanling *Dagla* and *Cnr1* KO mice at a time when their lean phenotype was developing. In addition, a pair-feeding study performed in weanling *Dagla* KO mice showed that hypophagia was the mechanism behind their lean phenotype, a result identical to that reported for a pair-feeding study of weanling *Cnr1* KO mice (17). The important role of hypophagia in development of the lean phenotype in *Dagla* and *Cnr1* KO mice suggests that the lean phenotype results primarily from loss of Dagla-generated 2-AG signaling through Cnr1 expressed by CNS rather than by peripheral tissues. This hypothesis is consistent with the observations that: (1) Cnr1 and Dagla are most highly expressed in the CNS, (2) brain 2-AG levels are much lower in *Dagla* KO mice compared to *Daglb* KO mice, and (3) removal of Cnr1 from neurons known to regulate energy balance, but not from non-neuronal peripheral tissues, is all that is required to reproduce the lean phenotype observed in global *Cnr1* KO mice [(32, 33, 42, 43); our unpublished observations]. Interestingly, hypophagia was not as pronounced in older *Dagla* KO mice, and in fact pair-feeding of WT mice to *Dagla* KO mice was not associated with marked loss of BW relative to ad lib-fed WT littermates, a finding that exactly reproduced results reported for pair-feeding studies using older *Cnr1* KO mice (17). Such results may simply reflect increased food intake as an adaptation to maintain already depleted body fat stores and LBM in adult *Dagla* and *Cnr1* KO mice, or they could suggest that food intake-independent mechanisms were operative in adult KO mice. However, activity was not increased in either our adult *Dagla* or *Cnr1* KO mice in three independent assays and there was no evidence of malabsorption in our *Dagla* KO mice. Also, VO_2_ was not increased in our *Dagla* KO mice or in *Cnr1* KO mice studied by others (17); however, the error inherent in the indirect calorimetry method and the ability of Cnr1 inverse agonists to increase energy expenditure in rodents and humans (44, 45) suggest that additional studies are needed to determine if increased energy expenditure contributes to the lean phenotype of older *Dagla* KO mice.

A number of metabolic parameters were studied in *Dagla* and *Cnr1* KO mice. Neither HFD-fed *Dagla* nor *Cnr1* KO mice showed consistent improvements in fasting blood glucose levels or glucose excursions during OGTTs, but both KOs had significantly lower insulin levels during a fast and 30 min after glucose challenge, along with lower triglyceride (TG) and total cholesterol levels. These results are similar to *Cnr1* KO mouse data published by others (20, 42, 43, 46). *Dagla* KO mice-fed western diet also showed a decrease in hepatic steatosis, which mirrored their decrease in total body fat. Past studies initially suggested that hepatic *Cnr1* deficiency alone prevented the HFD-induced development of hepatic steatosis, insulin resistance, and dyslipidemia, whereas extra-hepatic *Cnr1* deficiency was required to prevent HFD-induced obesity (46). Although subsequent studies reported that CNS-specific *Cnr1* deficiency alone prevented HFD-induced hepatic steatosis, insulin resistance, and dyslipidemia (42, 43), recent data indicate that peripherally restricted Cnr1 antagonists can indeed prevent this metabolic syndrome (47). Our data suggest that, wherever the Cnr1 that confers each of these effects is expressed, Dagla must also be expressed to provide the 2-AG that serves as the EC signal.

Mice treated with rimonabant show the same resistance to hepatic steatosis, insulin resistance, dyslipidemia, and obesity as do mice lacking *Cnr1* (19, 20, 42, 43, 46, 48) or *Dagla* (data presented here), suggesting that Dagla inhibitors may achieve the same effects as Cnr1 inverse agonists if delivered to the right location. In obese humans with T2D, rimonabant significantly lowered BW, A1C, insulin resistance, and serum TGs (22, 49), suggesting that Dagla inhibitors will also be effective in these individuals. Of the few Dagla inhibitors studied, the most promising data are for O-7460, a small molecule that lowered food intake in a dose-dependent manner during the 14 h after a single intraperitoneal injection; the highest dose was associated with slight but significant decreases in BW and hypothalamic 2-AG levels (50). Unfortunately, hypophagia is a common sign of off-target toxicity for many compounds. For this reason, developers of the Cnr1 inverse agonists, rimonabant and taranabant, demonstrated that their compounds lowered food intake in WT but not *Cnr1* KO mice (19, 51). Dagla inhibitors should also be tested in *Dagla* KO mice early in preclinical development to confirm that their effects on food intake are truly on-target.

The EC system plays an important role in regulating emotional behavior (26). Although the Cnr1 inverse agonist rimonabant was an effective anti-obesity agent, it was often associated with affective disturbances such as anxiety and depression that made it unsuitable for routine use (22, 24, 25). The value of Dagla as an anti-obesity target would be enhanced if the favorable metabolic phenotype shared by *Dagla* and *Cnr1* KO mice was not linked to the undesirable neuropsychiatric effects associated with rimonabant. For this reason, we chose to evaluate *Dagla* and *Cnr1* KO mice in a series of tests designed to evaluate anxiety, depression, and other behaviors. Interestingly, published studies that used these behavioral tests to evaluate Cnr1 KO mice, or WT mice treated with compounds that modulate Cnr1 signaling, reported results that were often inconsistent with each other and with data we collected on our Cnr1 KO mice (39, 40, 52–61). This suggested that the value in our behavioral screen would come from directly comparing the behavioral phenotypes of *Dagla* and *Cnr1* KO mice obtained in our laboratory. We found that *Dagla* and *Cnr1* KO mice differed only in the open-field time in center and the platform tests, two related tests that use the same open-field chamber; here *Dagla* KO mice exhibited a more anxiolytic behavior. *Dagla* and *Cnr1* KO mice showed similar behaviors in the hot plate, marble burying, open-field rearing, forced swim, open-field distance traveled, and tail suspension tests; for the first four of these tests, the behavior of KO mice from both lines appeared different from WT. The fact that both *Dagla* and *Cnr1* KO mice responded similarly in most of the tests suggests that the 2-AG synthesized by Dagla is mediating not only effects on pathways regulating food intake and various metabolic parameters but also effects on pathways that influence important neuropsychiatric behaviors. Our studies were limited by the fact that we only employed a few acute assays to assess depression and anxiety (62). In addition, the open-field time in center and platform tests suggest the possibility that the two KO lines may differ in anxiety-related behavior, with *Dagla* KO mice exhibiting a more anxiolytic response; this is a potentially important finding, because it raises the possibility that Dagla KO mice do not share an undesirable behavioral phenotype present in Cnr1 KO mice. Additional studies are therefore needed; if they ultimately confirm our overall impression that *Dagla* and *Cnr1* KO mice share behavioral phenotypes, the response to Dagla inhibitors may well be similar to the response to Cnr1 antagonists and inverse agonists for both metabolic and neuropsychiatric effects. Finally, we observed seizure activity followed by death in three *Dagla* KO mice; this may explain the decreased survival of our *Dagla* KO line, which occurs despite a lack of histopathologic findings. Although we did not assess survival of our *Cnr1* KO mice, others reported increased seizure activity and decreased survival without an obvious cause of death in *Cnr1* KO mice (21). These shared findings provide further evidence for a close interaction between Dagla and Cnr1 in the normal regulation of multiple neurologic pathways.

Our high-throughput phenotypic screen correctly identified the lean phenotypes of *Cnr1* and *Dagla* KO mice, just as it correctly identified the body fat phenotype of many KO lines in past studies; some of these KOs share their phenotype with humans having inactivating mutations in the same gene (30, 35, 63). Our screen has also identified genes that play a key role in regulating bone mass (31). Continued mining of our KO data, and KO data generated by the International Mouse Knockout Consortium, should identify genes that, when mutated, (1) result in specific diseases and (2) can be targeted to develop therapeutics for obesity, diabetes, osteoporosis, and other indications.

In summary, our data suggest that, in pathways where ECs signal through Cnr1 to regulate food intake, certain other behaviors and also other aspects of the metabolic phenotype observed in *Cnr1* KO mice, Dagla alone provides the 2-AG that serves as the EC signal. Based on these data, small molecule Dagla inhibitors with a pharmacokinetic profile similar to that of Cnr1 inverse agonists are likely to mirror the ability of these inverse agonists to decrease food intake, BW, TGs, and insulin resistance while improving glycemic control in obese patients with diabetes. Whether Dagla inhibitors will also be associated with undesirable neuropsychiatric events similar to those observed with use of Cnr1 inverse agonists, which must be considered possible based on data presented here, can only be answered with human clinical trials.

## Author Contributions

DP, KS, TL, ZD, UD, and BZ made substantial contributions to the conception or design of the work. DP, KS, UD, JG, NW, DD, RR, PV, GH, RB, and ZD made substantial contributions to the acquisition, analysis, or interpretation of data for the work. DP, ZD, and UD participated in drafting the work. JG, NW, DD, KS, TL, RR, PV, GH, RB, and BZ participated in revising it critically for important intellectual content. All authors provided final approval of the version to be published. All authors agree to be accountable for all aspects of the work in ensuring that questions related to the accuracy or integrity of any part of the work are appropriately investigated and resolved.

## Conflict of Interest Statement

All authors were employed by Lexicon Pharmaceuticals, Inc. at the time these studies were performed, and received compensation in the form of salary and stock options.

## Supplementary Material

The Supplementary Material for this article can be found online at http://www.frontiersin.org/Journal/10.3389/fendo.2015.00086/abstract
