# Supplementary material for: Diacylglycerol Lipase α Knockout Mice Demonstrate Metabolic and Behavioral Phenotypes Similar to Those of Cannabinoid Receptor 1 Knockout Mice
Source: Front Endocrinol (Lausanne). 2015 Jun 2;6:86. doi: 10.3389/fendo.2015.00086 (PMC4451644; doi:10.3389/fendo.2015.00086)
Supplement: Supplementary file 1 [file datasheet_1.pdf]

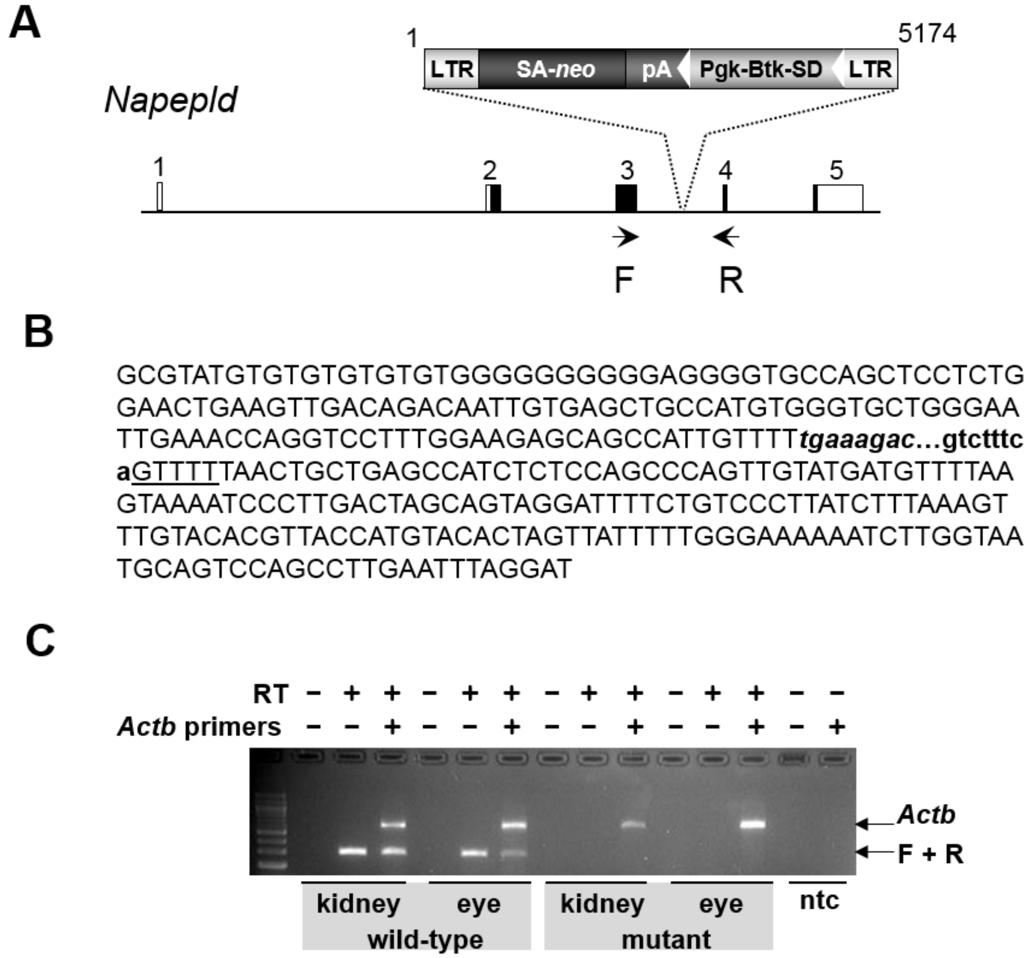

Figure S1. Generation of *Napepld* KO mice. **A**) Schematic diagram of the *Napepld* gene showing the location of retroviral gene-trap vector insertion in Omnibank embryonic stem (ES) cell clone OST429065 (accession CG656460.1). This clone was generated using vector VICTR48 (accession EU676804.1) which disrupts endogenous gene transcription through the action of a splice acceptor (SA-neo) cassette. Open boxes denote untranslated exons, filled boxed denote coding exons. LTR, viral long terminal repeat; SA, splice acceptor sequence; *neo*, neomycin phosphotransferase genes; pA, polyadenylation sequence; *Pgk*, phosphoglycerate kinase-1 promoter; *Btk*-SD, Bruton's tyrosine kinase splice donor sequence. Oligonucleotide primers (F and R) used to assess the efficiency of transcript capture are depicted with arrowheads. **B**) Mouse genomic sequence obtained by inverse PCR amplification of the proviral insertion site. Vector sequence is shown in lower case **bold** text, and is abbreviated. A five-base duplication of genomic sequence was observed at site of insertion, and is underlined. **C**) RT-PCR expression analysis of *Napepld* transcript. Endogenous *Napepld* transcript was detected in the kidney and eye of wild-type (+/+) mice but not in homozygous mutant (-/-) tissues. Primers F: 5'-CGTCTTCACGCCTTCCCAGCACT-3' and R: 5'-GAATCGCCACAGATCTCTTTGTTT GA-3' are complementary to *Napepld* exons 3 and 4, respectively, and amplify a product of 290 nucleotides. RT-PCR analysis using primers (*Actb*) complimentary to the mouse *beta actin* gene (accession number M12481) was performed in the same reaction as an internal amplification control. Lanes labeled "ntc" represent control reactions containing no template.

Table S1. Body composition of *Dagla* KO and WT mice by QMR at 16 weeks of age

Cohort	Genotype	N	Diet	Body weight (g)	Body fat (g)	% Body fat	LBM (g)
1-M	WT	16	Chow	31.3 ± 2.8	4.4 ± 1.7	13.9 ± 4.6	26.9 ± 2.1
1-M	KO	8	Chow	26.5 ± 1.7***	2.9 ± 1.0*	11.1 ± 4.2	23.6 ± 2.4**
1-F	WT	16	Chow	23.3 ± 2.4	4.0 ± 1.4	17.0 ± 5.0	19.3 ± 1.8
1-F	KO	9	Chow	20.2 ± 1.4**	2.5 ± 0.9**	12.2 ± 3.7*	17.7 ± 0.9*
2-M	WT	18	HFD	39.7 ± 5.2	11.8 ± 3.6	29.2 ± 5.6	27.9 ± 2.1
2-M	KO	9	HFD	32.5 ± 5.0**	7.9 ± 4.0*	23.2 ± 7.9*	24.6 ± 1.4***
2-F	WT	9	HFD	27.6 ± 3.7	8.7 ± 3.0	30.8 ± 7.0	18.9 ± 1.6
2-F	KO	11	HFD	22.2 ± 1.5***	4.0 ± 1.0***	17.9 ± 4.0***	18.2 ± 1.2
3-M	WT	13	HFD	38.8 ± 4.7	10.1 ± 3.5	25.6 ± 6.9	28.7 ± 2.8
3-M	KO	10	HFD	28.3 ± 3.6***	4.6 ± 2.7***	15.7 ± 7.5**	23.6 ± 1.8***
3-F	WT	11	HFD	26.9 ± 3.2	8.2 ± 2.6	30.1 ± 6.5	18.7 ± 1.3
3-F	KO	10	HFD	20.0 ± 3.3***	4.0 ± 1.9***	19.5 ± 5.9***	16.0 ± 2.0**
5-M	WT	15	HFD	39.7 ± 7.4	8.4 ± 4.5	20.1 ± 7.0	31.3 ± 3.3
5-M	KO	13	HFD	29.8 ± 3.3***	3.1 ± 0.5***	10.5 ± 1.7***	26.7 ± 3.2***
5-F	WT	12	HFD	29.0 ± 3.0	6.7 ± 2.4	22.6 ± 6.1	22.3 ± 0.9
5-F	KO	8	HFD	24.6 ± 1.7**	3.9 ± 0.9**	15.8 ± 3.1**	20.7 ± 1.2**
6-M	WT	14	HFD	34.8 ± 4.1	9.8 ± 2.7	28.4 ± 7.5	25.0 ± 4.2
6-M	KO	12	HFD	27.6 ± 2.6***	4.6 ± 1.0***	16.6 ± 3.0***	23.0 ± 2.2
6-F	WT	16	HFD	29.8 ± 4.2	8.9 ± 0.3	29.1 ± 6.8	20.9 ± 1.7
6-F	KO	15	HFD	23.7 ± 2.8***	5.0 ± 1.6***	20.8 ± 4.3***	18.7 ± 1.8**
7-M	WT	6	HFD	47.8 ± 7.0	15.5 ± 4.1	31.9 ± 5.2	32.4 ± 3.9
7-M	KO	10	HFD	33.1 ± 4.3***	6.8 ± 3.0***	20.0 ± 6.4**	26.2 ± 1.7***
7-F	WT	15	HFD	30.1 ± 5.5	9.3 ± 3.9	29.8 ± 9.3	20.9 ± 2.8
7-F	KO	8	HFD	23.3 ± 3.1**	4.7 ± 2.0**	19.4 ± 6.0*	18.6 ± 1.5
8-F	WT	8	Chow	24.0 ± 2.5	4.3 ± 1.0	17.6 ± 3.0	19.7 ± 1.7
8-F	KO	8	Chow	20.6 ± 2.3*	2.9 ± 0.7*	14.3 ± 2.8*	17.6 ± 1.9*

Data presented as mean ± SD; N = number of mice; M = Male, F = female; LBM, lean body mass.
KO different from WT, *P < 0.05; ** P < 0.01; ***P < 0.001.

Table S2. Body composition by DEXA of *Dagla* KO and WT mice aged 29 weeks

Cohort	Genotype	N	Diet	Body weight (g)	% Body fat	LBM (g)	BMD (mg/cm^2)
3-M	WT	13	HFD	47.4 ± 6.5	35.0 ± 7.2	30.0 ± 3.0	0.060 ± 0.003
3-M	KO	10	HFD	32.0 ± 6.0***	22.6 ± 8.3**	23.2 ± 2.0***	0.055 ± 0.003**
3-F	WT	11	HFD	34.0 ± 8.6	36.4 ± 13.5	20.4 ± 1.9	0.053 ± 0.003
3-F	KO	9	HFD	23.2 ± 4.8**	25.3 ± 8.4*	16.4 ± 1.8***	0.048 ± 0.004**

Data presented as mean ± SD; N = number of mice; M = Male, F = female; LBM, lean body mass; BMD, bone mineral density. KO different from WT, *P < 0.05; ** P < 0.01; ***P < 0.001.

Table S3. Body composition by QMR of adult *Cnr1* KO and WT mice

Cohort	Genotype	Age (wks)	N	Diet	Body weight (g)	Body fat (g)	% Body fat	LBM (g)
1-M	WT	16	15	HFD	37.5 ± 4.2	12.0 ± 3.6	31.6 ± 7.4	25.5 ± 2.6
1-M	KO	16	14	HFD	30.8 ± 3.7***	6.7 ± 2.5***	21.1 ± 5.3***	24.1 ± 1.5
1-F	WT	16	11	HFD	25.0 ± 2.5	7.0 ± 2.6	27.6 ± 8.1	18.0 ± 1.3
1-F	KO	16	10	HFD	23.0 ± 3.0	5.2 ± 2.0	22.1 ± 5.8	17.8 ± 1.5
2-F	WT	16	14	Chow	26.7 ± 4.4	5.1 ± 2.8	18.2 ± 6.1	21.6 ± 1.8
2-F	KO	16	9	Chow	22.4 ± 1.6*	2.9 ± 0.4*	12.8 ± 1.8*	19.5 ± 1.4**
3-M	WT	32	15	HFD	48.6 ± 7.1	15.8 ± 4	32.1 ± 5.5	32.8 ± 4.1
3-M	KO	32	9	HFD	32.8 ± 3.1***	6.5 ± 2.1***	19.8 ± 5.5***	26.2 ± 2.6***
3-F	WT	32	11	HFD	36.2 ± 5	12.9 ± 3.3	35.1 ± 4.6	23.3 ± 1.9
3-F	KO	32	7	HFD	28.8 ± 5.8*	6.2 ± 3.2***	20.7 ± 5.8***	22.6 ± 2.9
5-M	WT	16	8	HFD	32.7 ± 4.1	8.4 ± 2.7	25.3 ± 5.6	24.2 ± 2.0
5-M	KO	16	8	HFD	26.7 ± 3.4*	5.0 ± 1.8*	18.4 ± 4.0*	21.6 ± 1.8*
6-F	WT	21	8	Chow	26.3 ± 3.1	5.8 ± 2.3	21.5 ± 6.1	20.5 ± 1.3
6-F	KO	21	7	Chow	22.6 ± 2.0*	3.2 ± 0.5*	14.0 ± 2.3**	19.4 ± 2.0
7-M	WT	28	7	Chow	37.8 ± 5.3	7.7 ± 4.0	19.7 ± 7.6	30.1 ± 1.9
7-M	KO	28	7	Chow	28.4 ± 3.4**	3.1 ± 0.8*	10.7 ± 2.1*	25.4 ± 2.9**
7-F	WT	28	13	Chow	26.5 ± 5.6	5.8 ± 4.3	20.2 ± 9.4	20.7 ± 1.9
7-F	KO	28	9	Chow	24.0 ± 2.9	3.5 ± 0.8	14.5 ± 2.1	20.5 ± 2.2

Data presented as mean ± SD; N, number of mice; wks, weeks; M, male; F, female; LBM, lean body mass.
KO different from WT, *$P < 0.05$; ** $P < 0.01$; ***$P < 0.001$.

Table S4. Serum triglyceride and total cholesterol levels in *Dagla* and *Cnr1* KO mice

Cohort	Genotype	Age	N	Diet	TG (mg/dL)	Chol (mg/dL)
3-M	*Dagla* WT	15 mo	10	HFD	107.6 ± 44.0	250.8 ± 111.5
3-M	*Dagla* KO	15 mo	8	HFD	84.5 ± 41.7	147.0 ± 32.1*
3-F	*Dagla* WT	16 mo	9	HFD	100.1 ± 21.2	107.4 ± 33.2
3-F	*Dagla* KO	16 mo	6	HFD	87.3 ± 44.1	108.5 ± 26.5
4-M	*Dagla* WT	10 mo	12	HFD	109.3 ± 34.7	157.8 ± 42.1
4-M	*Dagla* KO	10 mo	5	HFD	71.8 ± 25.3*	158.6 ± 18.2
4-F	*Dagla* WT	9 mo	13	HFD	100.5 ± 25.7	131.9 ± 29.8
4-F	*Dagla* KO	9 mo	7	HFD	82.6 ± 21.2	118.3 ± 24.0
5-M	*Dagla* WT	4 mo	15	HFD	156.2 ± 46	150.6 ± 31.5
5-M	*Dagla* KO	4 mo	13	HFD	112.0 ± 24.7**	132.5 ± 30.0
5-F	*Dagla* WT	5 mo	12	HFD	129.5 ± 22	133.8 ± 30.4
5-F	*Dagla* KO	5 mo	8	HFD	88.9 ± 15.6***	121.5 ± 23.3
1-M	*Cnr1* WT	5 mo	15	HFD	196.3 ± 48.7	242.3 ± 82.5
1-M	*Cnr1* KO	5 mo	12	HFD	114.8 ± 51.1***	190.1 ± 38.0
1-F	*Cnr1* WT	5 mo	11	HFD	81.7 ± 13.7	167.8 ± 32.6
1-F	*Cnr1* KO	5 mo	9	HFD	75.2 ± 21.5	137.4 ± 26.1*

Data presented as mean ± SD; N = number of mice; TG, triglyceride; Chol, total cholesterol
KO different from WT, *$P < 0.05$; ** $P < 0.01$; ***$P < 0.001$.
